# Research status and hot topics of the effects of skin innervation on wound healing from 1959 to 2022: A bibliometric analysis

**DOI:** 10.3389/fsurg.2022.966375

**Published:** 2022-10-11

**Authors:** Ru Song, Zhenjie Wu, Jiaxu Ma, Siyuan Yin, Chunyan Liu, Rui Sun, Guoqi Cao, Yongpan Lu, Aoyu Chen, Guang Zhang, Jian Liu, Yibing Wang

**Affiliations:** ^1^ Department of Plastic Surgery, Shandong Provincial Qianfoshan Hospital, Shandong University, Jinan, China; ^2^ Jinan Clinical Research Center for Tissue Engineering Skin Regeneration and Wound Repair, The First Affiliated Hospital of Shandong First Medical University / Shandong Provincial Qianfoshan Hospital, Jinan, China; ^3^ Department of Plastic Surgery, The First Affiliated Hospital of Shandong First Medical University / Shandong Provincial Qianfoshan Hospital, Jinan, China

**Keywords:** skin innervation, wound healing, bibliometrics, VOSviewer, Citespace, WoSCC

## Abstract

**Background:**

Skin innervation plays an important role in wound healing by either direct contact with or indirect secretions that impact skin cells. Many studies in this field have been published; however, there is a lack of bibliometric analyses focusing on the effect of skin innervation on skin wound healing. In this study, we aimed to analyse the research trends, status, and hotspots in this field.

**Methods:**

Reviews and articles published in English were extracted from the Web of Science Core Collection (WoSCC) database based on subject term searches. Microsoft Office Excel, VOSviewer, and CiteSpace were used to analyse publication date, country or region, institution, author, and author keywords.

**Results:**

A total of 368 papers published between 1959 and 2022 were included in the analysis. Although there was a pulsation during this period, there was an overall upward trend in studies related to the effect of skin innervation on wound healing. The United States, particularly the *University of Washington*, and Gibran, Nicole S. from the *University of Washington*, was the most active in this field. *Wound Repair and Regeneration* published the most relevant literature, and “Calcitonin gene-related peptide: physiology and pathophysiology” had the highest total number of citations. “Diabetic foot ulcer,” “epidermal stem cells,” “mesenchymal stem cells,” and “mast cells” are current and potential future research hotspots.

**Conclusion:**

This bibliometric analysis will inform the overall trends in research related to the effect of skin innervation on wound healing, summarise relevant research hotspots, and guide future work.

## Introduction

Skin is the largest organ of the body, a barrier against attacks from the outside environment, and a powerful regulator of the stability of the inside environment. Therefore, the skin's normal wound healing process, including timely recovery of normal structure and function, is extremely important. Skin wound healing is a dynamic and complex process after skin injury that involves four spatially and temporally overlapping phases, namely, coagulation, inflammation, proliferation, and remodelling (1, 2), which are regulated by various factors, including skin innervation (3, 4). Cutaneous nerve fibres can regulate wound healing and maintain skin homeostasis by direct contact with skin cells, such as keratinocytes, fibroblasts, and vascular endothelial cells (5–8), or indirect secretion of nerve growth factors or neuropeptides (8, 9) to modulate skin cell functions. Skin regeneration cannot be achieved without neurogenic factor effects because nerves can regulate normal epithelialisation (10), angiogenesis (11), extracellular matrix remodelling (12), regeneration of skin appendages (13, 14), and sensation recovery (15). In contrast, denervation, such as spinal cord injury (16, 17), and peripheral nerve lesions, such as diabetic neuropathy (18) and deep burn wounds (15), can delay wound healing. Conversely, excessive nerve innervation may result in hypertrophic scars and keloids and uncomfortable sensations, such as itching and pain (19, 20). Many researchers have studied and published papers on the effect of skin innervation on wound healing. To better understand the research status of and hot topics in this domain, it is necessary to comprehensively analyse the current data from related research.

Bibliometric analysis is a novel scientific information visualisation method to quantitatively and qualitatively analyse the literature, including country, institution, author, journal, keywords, and reference data, thereby helping obtain an overview of the bibliometric characteristics of the literature and discover up-and-coming topics (21–23). Commonly used analysis tools include software such as HistCite (24), VOSviewer (25), and CiteSpace (26, 27) and online analysis platforms such as http://bibliometric.com/. To the best of our knowledge, no bibliometric studies have explored skin innervation effects on wound healing.

In this article, integrated bibliometric approaches were applied to perform a bibliometric and visual analysis of the literature related to the effect of skin innervation on wound healing published through 23 July 2022. The results reveal the research trends, hotspots, and frontiers of this field and will provide more understanding of past research and guide future studies.

## Materials and methods

### Data source and search strategy

The Web of Science Core Collection (WoSCC) database was used to retrieve all the published literature through the library website of Shandong University. Literature with publication dates through the beginning to 2022 was collected. The search terms were as follows: “TS = [(skin OR cutaneous) AND (“wound healing” OR “wound repair” OR “wound regeneration” OR “wound closure” OR wound OR scar OR burn OR “diabetic foot ulcer*”)] AND TS = (*innervat* OR neuropeptide* OR neurotransmitter* OR NGF OR “neural factor*” OR nerve* OR neuron* OR nervous OR neurogenic OR neuroinflammation OR NTR OR CD271 OR *denervat* OR neuromediator* OR neurohormone*) AND LANGUAGE = (English) AND DOCUMENT TYPES = (Article OR Review)”.

### Data download and screening

**Figure 1:**
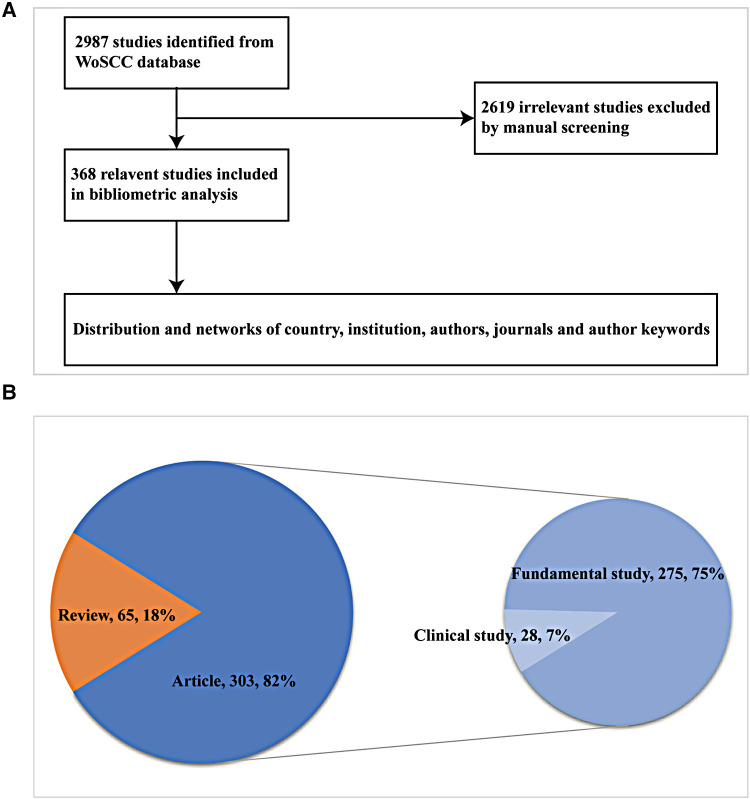
Data collection and screening process and results. (**A**) Flowchart detailing the paper collection and screening process. (**B**) Pie chart of the number and proportion of each type.

After searching with the abovementioned strategy on 23 July 2022, the full record and cited references of 2,987 publications were exported and downloaded as a tab delimited file, plain text file, and Excel file. Then, manual screening of the results of the primary research was independently performed by RSO and JM by viewing the title, abstract, author keywords, keywords plus, and full text to filter out irrelevant literature on the effect of skin innervation on wound healing. Ultimately, 2,619 papers were excluded and 368 studies included in the bibliometric analysis: 303 articles (82%) and 65 reviews (18%) (Figures 1A,B).

### Bibliometric analysis

The study applied Microsoft Office Excel 2019 to analyse the document distribution characteristics of type, publication year, and document citations through the exported “.xls” format files.

The “tab delimited” format file was imported into VOSviewer 1.6.18 (Leiden University, the Netherlands) (25) to summarise and analyse journal document counts and citations and visualise the distribution, co-authorship networks, and overlay networks of countries/regions, institutions (28–30), and authors and the co-occurrence of author keywords in default settings. The journal impact factors (IFs) were obtained from the 2021 Journal Citation Reports (JCR) to assess the scientific merit of a paper and journal (31, 32). For the two kinds of co-authorship maps, each node represents an item (countries/regions, institutions, authors, or author keywords), and the size of the node represents the document count of the countries/regions, institutions, or authors or the occurrences of author keywords. Each line represents a link between the above items, and its thickness represents the strength of the co-authorship or co-occurrence link. A cluster is a set of items included on a map, and in the network visualisation map, the colour of each item is determined by the cluster to which the item belongs. In the overlay visualisation map, the colour of each item represents the average publication year, according to the colour gradient presented in the lower right corner, with blue representing earlier publication years and yellow representing recent research.

The “plain text” format file was imported into CiteSpace 6.1.R2, 64 bits (Drexel University, Philadelphia, PA, United States) (26, 27), to identify author keywords with citation bursts, which is a computational technique for detecting sudden changes in author keyword citations and thus reflects hotspots within a time period. The settings for CiteSpace were the default settings, with a time range from January 1959 to December 2022 and 1 year per slice.

### Research ethics

All the data were downloaded from the WoSCC database without further animal and human experiments. No ethical approval was needed.

## Results

### Overview and analysis of the numbers of publications and citations

**Figure 2:**
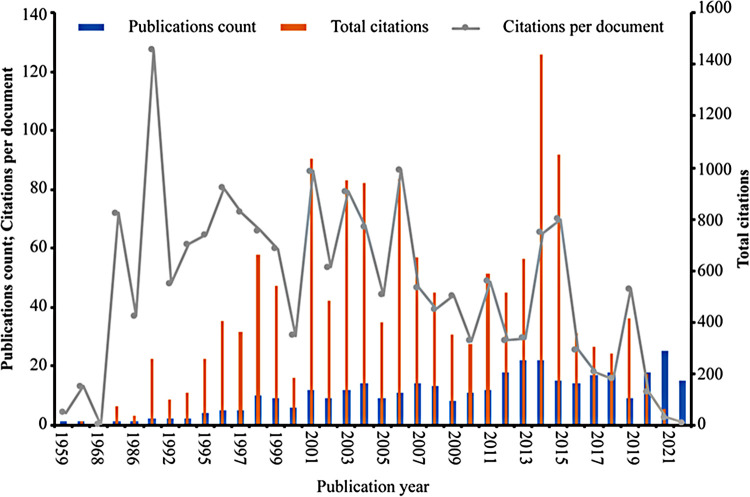
Number of annual publications, total citations, and average citations per document of the research. The blue bars represent the annual publication count, the orange bars represent the total number of annual citations, and the grey line represents the annual average number of citations per document.

As shown in Figures 1B, 2, Table 1, and Supplementary Table S1, the 368 included reviews and articles were cited a total of 15,453 times, with an average citation frequency of 42 for each publication. Of the 303 articles, only 28 clinical studies were published, while 275 fundamental studies focusing on cells, organoids, animals, and molecular techniques were published. The annual publication count, annual total citations, and annual citations per publication trends of the 64 years could be divided into four phases.

**Table 1:** The four-stage pattern of the effect of skin innervation on wound healing.

Stage (year)	Total counts	Review counts	Article counts		Total cites	Cites per docs
			Fundamental study	Clinical study		
1959–1990	5	0	5	0	126	25.2
1991–2006	112	16	86	10	7,637	68.2
2007–2014	120	19	91	10	5,014	41.8
2015–2022	131	30	93	8	2,676	20.4

Phase 1 occurred from 1959 to 1990, representing the initial stage of the research. In this 32-year period, which made up half of the total 64-year period, only five fundamental articles were published, with a total of 126 citations, and the number of citations per publication was 25.2. The first publication on the effect of skin innervation on wound healing published in 1959 (33) occurred during this phase, while the subsequent four were published separately in 1964 (34), 1968 (35), 1984 (36), and 1986 (37).

Phase 2 (1991–2006) lasted 16 years, half of the other 32 years after phase 1, with a rapidly increasing number of publications (112 studies), the highest total number of citations (7,637 citations), and the highest number of citations per publication (68.2 citations). The 112 publications of phase 2 were composed of 16 reviews and 96 articles, and the 96 articles contained 86 fundamental studies and 10 clinical studies. Among the years included in the analysis, 1991 had the highest average number of citations per publication (127.5 times), with two published fundamental studies (38, 39). The total number of citations for the 11 papers published in 2006 was 953, ranking the fourth highest, while the average number of citations per publication in 2006 was 87, ranking the second highest.

Phase 3 lasted from 2007 to 2014, a total of 8 years. The total publication count (120 studies) was 8 more than that of phase 2, with 19 reviews, 10 clinical research articles, and 91 fundamental research articles published, while the total number of citations (5,014 times) was only two-third of that in phase 2, and the number of citations per publication (41.8 times) was approximately half of that in phase 2. However, the number of total citations in 2014 was 1,437, which accounted for more than one-third of the total of phase 3 and was the highest of all the analysed years. The total number of publications in 2013 and 2014 was 22, ranking the second highest among the analysed years.

Phase 4 took place from 2015 to 2022 (the last 8 years). The total number of publications (131 studies) was slightly higher than that in phase 3. Notably, there were 30 reviews in this phase, while the article number was the same as that of phase 3, with 93 fundamental studies and 8 clinical studies. However, the total number of citations (2,676 times) and number of citations per publication (20.4 times) were approximately half of those in phase 3. Interestingly, the number of publications (25 studies) in 2021 was the highest in the 64 years, and the 2022 data may exceed it, as there were already 15 publications in the first 6 months of the year.

Overall, the above analysis revealed that research on the effect of skin innervation on wound healing has been intensive and is increasing.

### Analysis of productive journals

A total of 206 journals published at least one study on the effect of skin innervation on wound healing. Table 2 lists the top 10 journals publishing the greatest number of studies, covering 27.4% (101/368) of the related literature overall and representing the journals with the most acceptance of research in this field. *Wound Repair and Regeneration* (17 publications, 16.8%) ranks first, followed by the *Journal of Investigative Dermatology* and *Experimental Dermatology* (both 13 publications, 14.3%), and *Experimental Dermatology* had the highest total citations (691 times). Although only five related papers were published in *Proceedings of the National Academy of Sciences of the United States of America*, the journal ranked in the top 3 for the total number of citations (623 times) and first for the number of citations per document (124.6 times). The *British Journal of Dermatology* also had five publications but had the second-highest average number of citations per document (89.0 times), while the *International Journal of Lower Extremity Wounds* (five publications) had 0 citations. *Developmental Biology* had the second-highest total number of citations (677 times), and the average number of citations per document ranked third (67.7 times). In the top 10 most productive journal lists, more than 50% were from the United States, and Q1, Q2, and Q3 in JCR both accounted for one-third. The total publication count (46 studies) and total citations (1,948 times) of the Q2 journals ranked first, slightly higher than those of the Q1 journals (31 publications, 1,831 times) and substantially higher than those of the Q3 journals (24 publications, 657 times). The top two highest IFs (IF, 2021) on the list again belonged to the *Proceedings of the National Academy of Sciences of the United States of America* (Q1, IF = 12.779) and the *British Journal of Dermatology* (Q1, IF = 11.113), consistent with the ranking of citations per document.

**Table 2:** Top 10 journals that published research on skin innervation effects on wound healing.

No.^a^	Journal name	Counts (%)	Total cites	Cites per docs	IF (2021)	JCR	Country/region
1	*Wound Repair and Regeneration*	17 (16.8)	476	28.0	3.401	Q2	United Kingdom
2	*Journal of Investigative Dermatology*	13 (14.3)	533	41.0	7.59	Q1	United Kingdom
2	*Experimental Dermatology*	13 (14.3)	691	53.2	4.511	Q2	United Kingdom
4	*Developmental Biology*	10 (11.0)	677	67.7	3.148	Q2	United States
5	*Plastic and Reconstructive Surgery*	8 (8.8)	230	28.8	5.169	Q1	United States
6	*Cell and Tissue Research*	7 (7.7)	191	27.3	4.051	Q3	Germany
7	*Annals of Plastic Surgery*	6 (6.6)	119	19.8	1.763	Q3	United States
7	*Journal of Surgical Research*	6 (6.6)	347	57.8	2.417	Q3	United States
7	*PLoS One*	6 (6.6)	104	17.3	3.752	Q2	United States
10	*British Journal of Dermatology*	5 (5.5)	445	89.0	11.113	Q1	United Kingdom
10	*International Journal of Lower Extremity Wounds*	5 (5.5)	0	0.0	1.922	Q3	United States
10	*Proceedings of The National Academy of Sciences of The United States of America*	5 (5.5)	623	124.6	12.779	Q1	United States

^a^ The journals with the same number of publications are ranked in the same place.

IF, impact factor; JCR, Journal Citation Report.

### Analysis of the related documents with the most citations

Table 3 lists the top 10 documents on the effect of skin innervation on wound healing with the most total citations. Only one paper was published before 2000 (year 1998) (8), and zero paper was published in the past 5 years. A total of 40% of these papers were original articles focusing on stem cell and regeneration. The 10 papers were published in 9 journals, and 3 were on the top 10 productive journals list (Table 2, *Experimental Dermatology*, *British Journal of Dermatology*, and *Developmental Biology*), and 90% belonged to Q1 and Q2. “Calcitonin gene-related peptide: Physiology and pathophysiology” (publication year: 2014) (40) was the most frequently cited (529 times) and annually cited (58.8 times) publication. Annual citations were calculated by dividing the total number of citations by the total number of years since publication. “Mesenchymal stem cell exosomes induce proliferation and migration of normal and chronic wound fibroblasts, and enhance angiogenesis in vitro” (publication year: 2015) (41) ranked third in terms of the total citations (350 times) and second in terms of the annual citations (43.8 times).

**Table 3:** Top 10 most frequently cited documents on the effects of skin innervation on wound healing.

No.	Document title	Year	Type	Journal	Total cites	Annual cites
1	Calcitonin gene-related peptide: physiology and pathophysiology	2014	Review	*Physiology Reviews*	529	58.8
2	Neuronal control of skin function: the skin as a neuroimmunoendocrine organ	2006	Review	*Physiology Review*	372	21.9
3	Mesenchymal stem cell exosomes induce proliferation and migration of normal and chronic wound fibroblasts, and enhance angiogenesis in vitro	2015	Article	*Stem Cells and Development*	350	43.8
4	Cellular and molecular mechanisms of repair in acute and chronic wound healing	2015	Review	*British Journal of Dermatology*	312	39.0
5	Neuropeptides in the skin: interactions between the neuroendocrine and the skin immune systems	1998	Review	*Experimental Dermatology*	306	12.2
6	Nerve-derived sonic hedgehog defines a niche for hair follicle stem cells capable of becoming epidermal stem cells	2011	Article	*Cell Stem Cell*	295	24.6
7	Purinergic signalling	2006	Article	*British Journal of Pharmacological Science*	286	16.8
8	Pathogenesis and treatment of impaired wound healing in diabetes mellitus: new insights	2014	Review	*Advances in Therapy*	244	27.1
9	Glutamate signalling in non-neuronal tissues	2001	Review	*Trends in Pharmacological Sciences*	244	11.1
10	A stepwise model system for limb regeneration	2004	Article	*Developmental Biology*	228	12.0

### Analysis of active countries/regions and international collaboration

**Figure 3:**
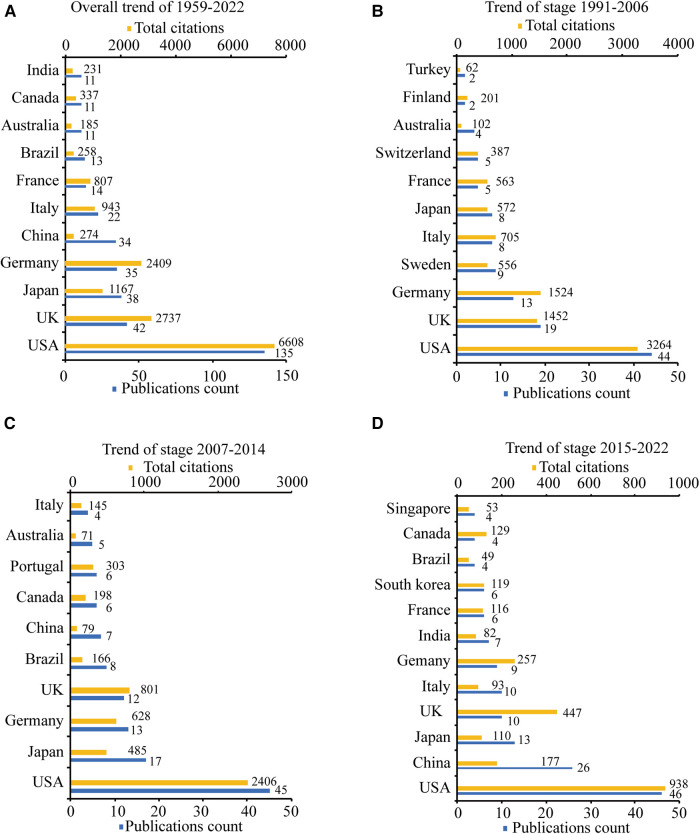
The top 10 productive countries/regions ordered by publications count. (**A**) The publications count and total citations of the top 10 countries/regions from 1959 to 2022. (**B**) The publications count and total citations of the top 10 countries/regions from 1991 to 2006. (**C**) The publications count and total citations of the top 10 countries/regions from 2007 to 2014. (**D**) The publications count and total citations of the top 10 countries/regions from 2015 to 2022.

A total of 47 countries or regions contributed to the research on the effect of skin innervation on wound healing. Figure 3 lists the top 10 productive countries or regions overall from 1959 to 2022 and the top 10 for each of the three periods (1991–2006, 2007–2014, and 2015–2022) in terms of publication counts in this field (papers with authors from different countries or regions appearing together are repeatedly counted). The United States ranked first, with 135 papers (37.1%) published, far more than the United Kingdom (42 papers, 11.5%), Japan (38 papers, 10.4%), Germany (35 papers, 9.6%), and China (32 papers, 8.8%) (Figure 3A). In all three periods (Figures 3B–D), the United States was always in first place, and the United Kingdom, Germany, Italy, Australia, and Japan were always active. The number of documents published by Brazil, Canada, and China started to increase only after 2007. China, in particular, was in second place, with 26 documents from 2015 to 2022. Regarding the number of citations, the United States (6,608 times) and the United Kingdom (2,737 times) ranked first and second, respectively, while Germany (2,409 times), exceeding Japan (1,167 times), came third, and China had only 256 total citations.

**Figure 4:**
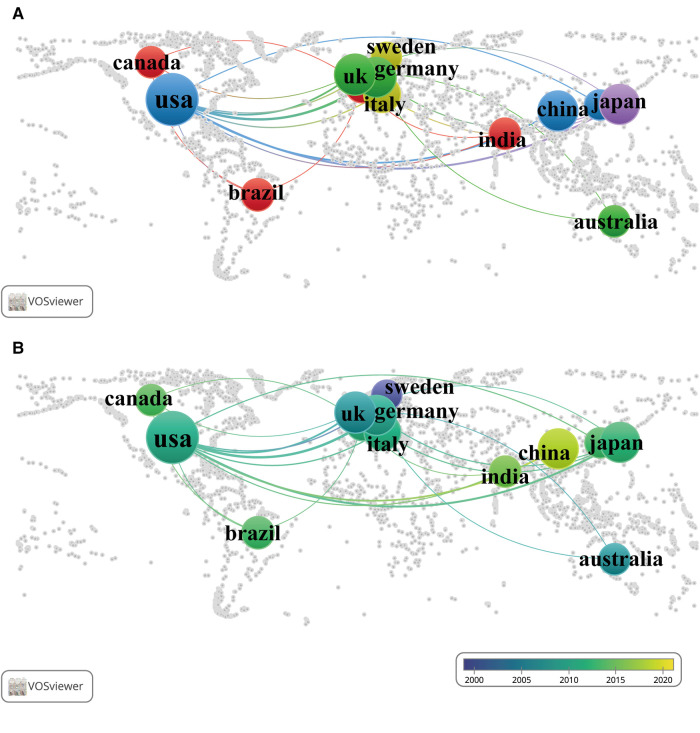
International collaboration networks among different countries/regions. Each node represents a country/region, and each line represents a link between two countries/regions. The size of each node represents the number of documents, and the thickness of each line represents the strength of the link. (**A**) The collaboration of 13 countries/regions with at least nine published documents on the world map. The colour of each node represents the clusters of each country/region. (**B**) The time-overlay collaboration map of 13 countries/regions with at least nine published documents on the world map. The colour of each node represents the average publication year, according to the colour gradient presented in the lower right corner.

As shown in Figure 4A and Supplementary Table S2, the country co-authorship network for the 13 countries with at least nine published documents was classified into five clusters automictically by VOSviewer, as indicated by five different colours. The United States was the country most actively involved in international collaboration, with the highest total link strength of 44, and had the most collaborations with German researchers (link strength = 13). The country co-authorship overlay in Figure 4B, together with Supplementary Table S2, shows that researchers of Sweden, Australia, and the United Kingdom (bright purple, with an average publication year: 2000.56, 2006.91, 2007.00) started work in this field in the early period, while researchers in China, India, Brazil, and Canada (bluish yellow, with an average publication year: 2017.55, 2015.00, 2013.00, and 2013.64) started their exploration in the latest period.

### Analysis of active institutions and cooperation

**Figure 5:**
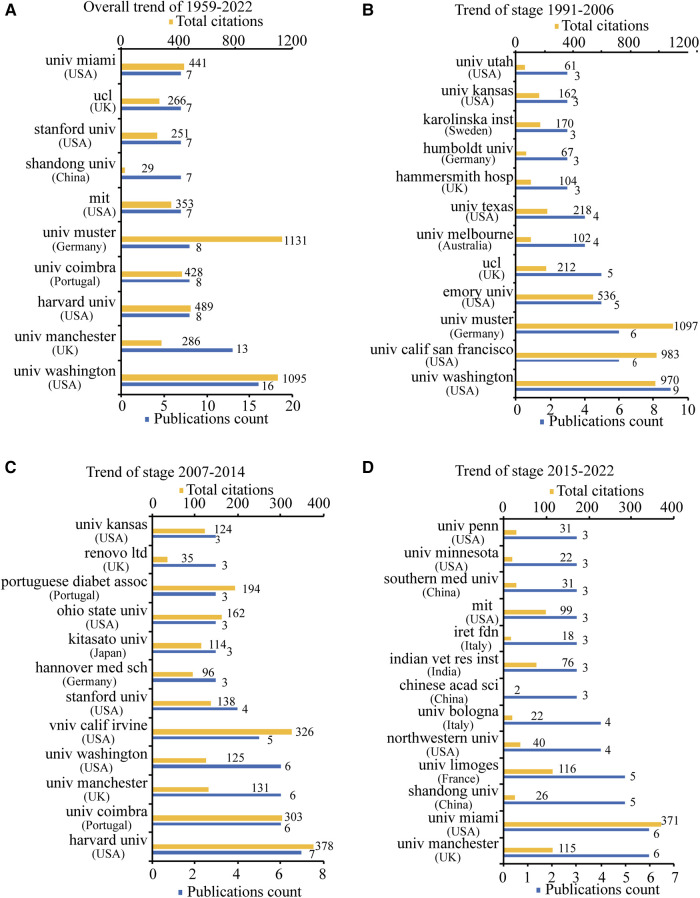
The top 10 productive institutions ordered by publications count. (**A**) The publications count and total citations of the top 10 institutions from 1959 to 2022. (**B**) The publications count and total citations of the top 10 institutions from 1991 to 2006. (**C**) The publications count and total citations of the top 10 institutions from 2007 to 2014. (**D**) The publications count and total citations of the top 10 institutions from 2015 to 2022.

**Figure 6:**
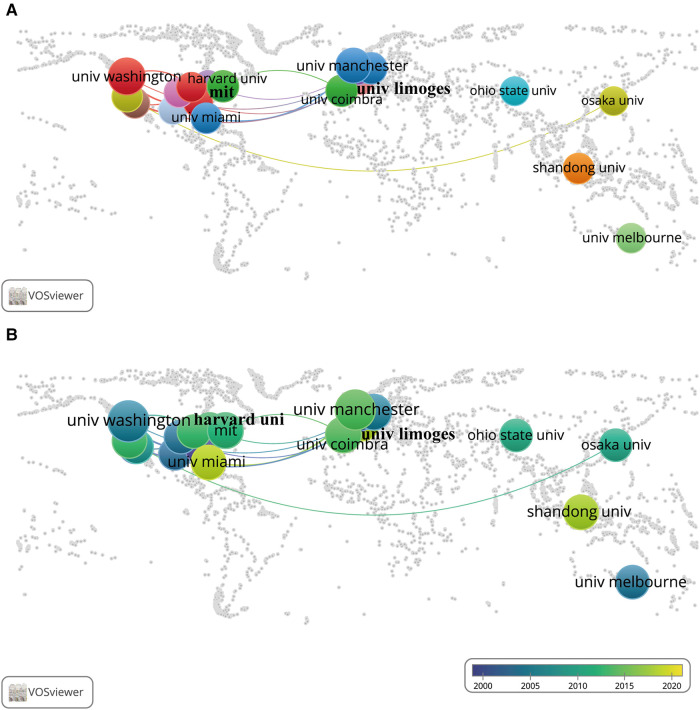
Institution cooperation networks. Each node represents an institution, and each line represents a link between two institutions. The size of each node represents the number of documents, and the thickness of each line represents the strength of the link. (**A**) Institutional cooperation network map of institutional co-authorship among the top 21 most productive institutions. The colour of each node represents the clusters of each institution. (**B**) Institutional co-authorship time-overlay map of cooperation networks among the top 21 most productive institutions. The colour of each node represents the average publication year, according to the colour gradient presented in the lower right corner.

A total of 519 institutions published studies on skin innervation effects on wound healing, and the overall top 10 of 1959–2022 and top 10 for each of the three periods (1991–2006, 2007–2014, 2015–2022) are listed in Figure 5 according to the number of publications. Half of the institutions are in the United States, and the *University of Washington* in the United States ranked first, with 16 publications, while the *University of Munster* in Germany also ranked first, with 1,131 total citations (Figure 5A). Shown in three periods in Figures 5B–D, the largest proportion of documents in all three periods was from American institutions, but the proportion decreased overall [from 58.3% (7/12) in the first period to 50% (6/12) in the second period to 38.5% (5/14) in the most recent period]. An analysis of the institutional co-authorship network (Figure 6A, Supplementary Table S3) revealed that cooperative relationships among institutions were dispersed. The 21 institutions were classified into 12 clusters, and the largest cluster included four institutions: the *University of Washington*; the *University of California, San Francisco*; *Northwestern University*; and *Emory University*. The time-overlay visualisation in Figure 6B and Supplementary Table S3 and periods displayed in Figure 5D indicate that the *University of Limoges* (France), *Shandong University* (China), and *University of Miami* (United States) (both in bluish yellow) recently started research in this field, with average publication years of 2017.60, 2017.14, and 2017.86, respectively. In addition, as shown in Figure 5D, we found that the *University of Bologna* and *IRET Foundation*, two Italian institutions, recently published four and three documents, respectively, accounting for 70% of Italian publications from 2015 to 2022.

### Analysis of authors and co-authorship

**Figure 7:**
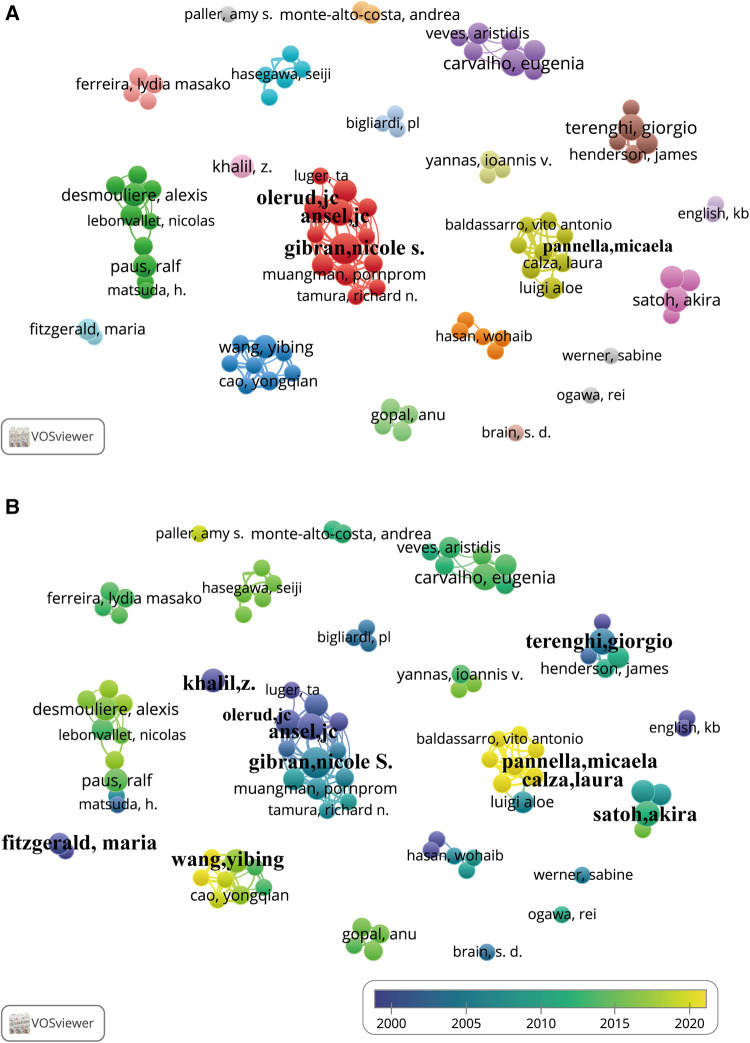
Author co-authorship networks. Each node represents an author, and each line represents a link between two authors. The size of each node represents the number of documents of each author, and the thickness of each line represents the strength of the link. (**A**) Co-authorship network map of the 90 authors with at least three documents. The colour of each node in (**A**) represents the clusters of each author. (**B**) Author co-authorship time-overlay map of the co-authorship networks of the 90 authors with at least three documents. The colour of each node represents the average publication year, according to the colour gradient presented in the lower right corner.

A total of 1,606 authors published at least one paper in this field, and the top 20 most productive authors in terms of publication count are listed in Table 4 and the top 5 in three periods (1991–2006, 2007–2014, and 2015–2022) are listed in Supplementary Table S4. Publications with more than one author were counted repeatedly by VOSviewer. Gibran, Nicole S. (11 papers, 8.5%, *University of Washington*, United States) was at the top of the list, followed by Ansel, John C. (8 papers, 6.2%, *Emory University*, United States), Carvalho, Eugenia (8 papers, 6.2%, *University of Coimbra*, Portugal), and Terenghi, Giorgio (8 papers, 6.2%, *The University of Manchester*, United Kingdom). Among authors from Japan, Australia, and China, only one from each country made the list: Satoh, Akira (7 times, *Okayama University*, Japan), Khalil, Zeinab G. (6 times, *The University of Melbourne*, Australia), and Wang Yibing (6 times, *Shandong University*, China). The total number of citations (1,009 times) and average number of citations per document (168.2 times) of Bunnett, Nigel W. (*University of California, San Francisco*, United States) was the highest, while Ansel, John C. (*Emory University*, United States) ranked second in both total citations (969 times) and average number of citations per document (121.1 times). The co-authorship networks for the 90 authors with at least 3 documents were grouped into 21 clusters, as shown in Figure 7A and Supplementary Table S5. The cooperative relationships among authors are distributed in a decentralised manner, and such cooperation was always limited to within the same country and institution. Gibran, Nicole S., Olerud, John E., and Ansel, John C., all from the United States, collaborated with each other the most, with total link strengths of 41, 31, and 27, respectively. The time-overlay co-authorship network in Figure 7B and the list in Supplementary Tables S4 and S5 indicate that Ansel, John C., Fitzgerald, Maria, and Khalil, Zeinab G. (bright purple) started research in this field at the beginning, with an average publication year of approximately 2000. Gibran, Nicole S., Terenghi, Giorgio, and Satoh, Akira (aquamarine blue, average publication year 2005) conducted their research from the initial to the middle stages, while Wang Yibing, Calza, Laura and Pannella, Micaela (icterine) published related articles in recent years.

**Table 4:** Top 20 authors that published documents on the effects of skin innervation on wound healing.

No.^a^	Author	Counts (%)	Total cites	Cites per docs	Countries/regions
1	Gibran, Nicole S.	11 (8.5)	524	47.6	United States
2	Ansel, John C.	8 (6.2)	969	121.1	United States
2	Carvalho, Eugenia	8 (6.2)	428	53.5	Portugal
2	Terenghi, Giorgio	8 (6.2)	198	24.8	United Kingdom
5	Olerud, John E.	7 (5.4)	743	106.1	United States
5	Satoh, Akira	7 (5.4)	346	49.4	Japan
7	Bunnett, Nigel W.	6 (4.6)	1,009	168.2	United States
7	Desmouliere, Alexis	6 (4.6)	159	26.5	France
7	Gardiner, David M.	6 (4.6)	554	92.3	United States
7	Khalil, Zeinab G.	6 (4.6)	170	28.3	Australia
7	Paus, Ralf	6 (4.6)	198	33.0	United States
7	Wang, Yibing	6 (4.6)	24	4.0	China
13	Bryant, Susan V.	5 (3.8)	520	104.0	United States
13	Ferguson, Mark W. J.	5 (3.8)	105	21.0	United Kingdom
13	Fitzgerald, Maria	5 (3.8)	285	57.0	United Kingdom
13	Luigi Aloe	5 (3.8)	391	78.2	Italy
13	Misery, Laurent	5 (3.8)	205	41.0	France
13	Moura, Liane I. F.	5 (3.8)	246	49.2	Portugal
13	Muangman, Pornprom	5 (3.8)	231	46.2	United States
13	Muffley, Lara A.	5 (3.8)	255	51.0	United States
13	Veves, Aristidis	5 (3.8)	621	124.2	United States

^a^ The authors with the same number of publications are ranked in the same place.

### Analysis of author keywords and author keyword co-occurrence

**Figure 8:**
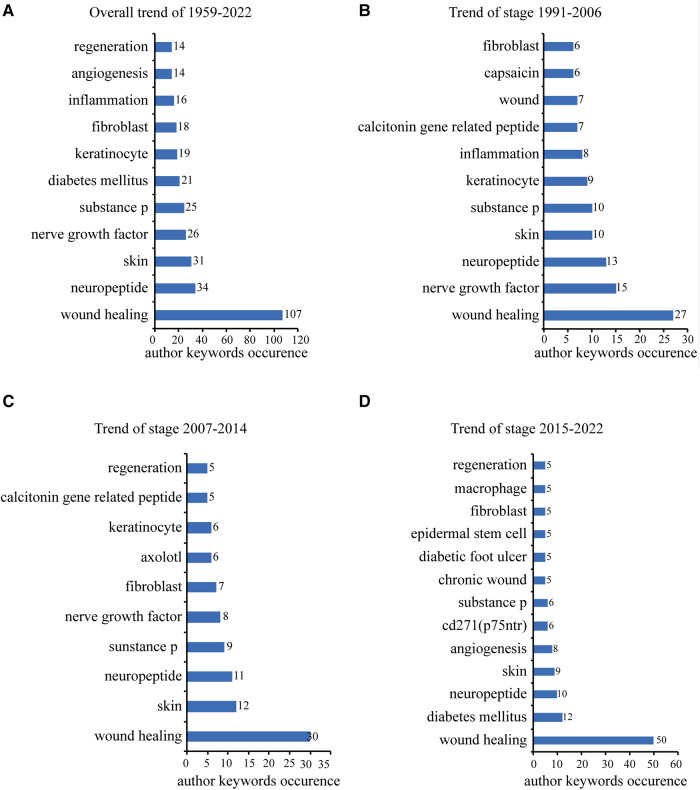
The top 10 frequently occurred author keywords ordered by occurrences. (**A**) Number of the top 10 frequently occurred author keywords 1959 to 2022. (**B**) Number of the top 10 frequently occurred author keywords from 1991 to 2006. (**C**) Number of the top 10 frequently occurred author keywords from 2007 to 2014. (**D**) Number of the top 10 frequently occurred author keywords from 2015 to 2022.

**Figure 9:**
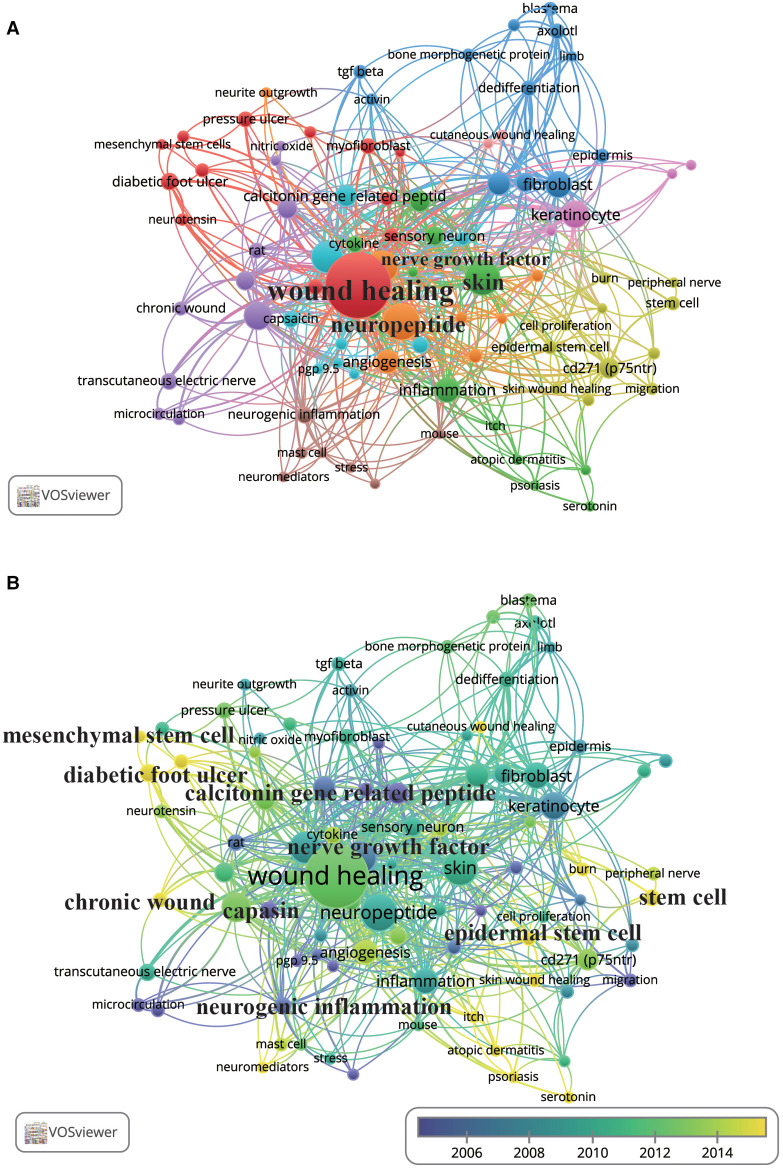
Co-occurrence networks of author keywords. Each node represents an author keyword, and each line represents a link between two author keywords. The size of each node represents the author keyword occurrence, and the thickness of each line represents the strength of the link. (**A**) Co-occurrence network map of the 88 author keywords that occurred at least three times. The colour of each node represents the clusters of author keywords. (**B**) Author keyword co-occurrence time-overlay map of the co-occurrence networks of the 88 author keywords that occurred at least three times. The colour of each node represents the average publication year, according to the colour gradient presented in the lower right corner.

Of 714 author keywords, a total of 88 occurred at least three times in the analysed articles. As shown in Figures 8A, 9A, and Supplementary Table S6, the author keywords were classified into 10 clusters, as indicated by different colours. “Wound healing” (107 occurrences) ranked first, with far more instances than “neuropeptide” and “skin”, with 34 and 31 occurrences, respectively. The three words were also the most co-occurring words and were all in the top 10 in the three periods (1991–2006, 2007–2014, and 2015–2022) (Figures 8B–D). The overlay visualisation network is displayed in Figure 9B, and the keywords are classified by colour in terms of the time of occurrence so that the colour can be used to represent the research history and identify research hotspots. “Calcitonin gene-related peptide” (2006.08), “capsaicin” (2002.14), “nerve growth factor” (2006.81), and “neurogenic inflammation” (2005.71), all in livid colour, were the early hotspots in the field. “Chronic wounds” and “diabetic foot ulcer” are shown in yellow in Figure 9B and frequently occurred in the 2015–2022 period depicted in Figure 8D, indicating that they have been hot topic diseases and conditions in recent years. Figures 8B,C show that in the first two phases (1991–2006, 2006–2014), “keratinocyte” appeared more frequently, but in more recent years (2015–2022, Figure 8D), “epidermal stem cells” appeared more frequently. “Mesenchymal stem cells,” “epidermal stem cells,” and “stem cells,” also in yellow in Figure 9B, have been studied recently and may still be hotspots in the future. Studies of the neuroimmunological effect of skin nerves on wound healing have flourished, as “neuromediators” and “mast cells” are a greenish yellow colour in Figure 9B, and “macrophages” began to be studied frequently in the 2015–2022 period (Figure 8D). Figures 8C,D also show that from 2007 to 2014, the words “axolotl” and “regeneration” began to appear more frequently and continued to do so until 2015–2022, meaning that the focus has gradually shifted from wound healing to wound regeneration.

The top 10 keywords with the strongest citation bursts, as shown in Table 5, were drawn and exported from CiteSpace (6.1.R2). “Nerve growth factor” had the highest burst strength (5.46), followed by “angiogenesis” (5.12) and “mesenchymal stem cell” (3.71). The burst interval of “mesenchymal stem cell”and “angiogenesis” lasted the longest, while “diabetic foot ulcer” lasted from 2019 to 2022, indicating current and future research hotspots.

**Table 5:** Top 10 author keywords with the strongest citation bursts.

Author keywords	Year	Strength	Begin	End	1959–2022
Nerve growth factor	1959	5.46	2003	2006	——————————————————
Angiogenesis	1959	5.12	2017	2022	——————————————————
Mesenchymal stem cell	1959	3.71	2015	2020	——————————————————
Innervation	1959	3.74	1994	1998	——————————————————
Microvascular endothelial cell	1959	3.41	2006	2009	——————————————————
Inflammation	1959	3.00	2014	2015	——————————————————
Human keratinocyte	1959	2.95	1998	2002	——————————————————
Diabetic foot ulcer	1959	2.65	2019	2022	——————————————————
Regeneration	1959	2.51	2018	2019	——————————————————
Epidermal stem cell	1959	2.30	2016	2018	——————————————————

## Discussion

As a niche discipline compared with psoriasis, atopic dermatitis (42, 43), the study of the effect of skin innervation on wound healing, has undergone long and slow development from 1959 to the present day, with many researchers from many institutions in many countries publishing their findings in a variety of journals. In this article, for the first time, we use bibliometric methods to analyse these findings, map the relationship network, summarise research trends and hotspots, and provide guidance for future research.

*Wound Repair and Regeneration* has published the most relevant literature, indicating that the journal itself has a preference for research in this area, suggesting that it could be a good choice as a platform to publish relevant research findings. *Experimental Dermatology* (Q2) had the highest number of total citations, while *Proceedings of the National Academy of Sciences of the United States of America* (Q1) had the highest number of average citations per document and the highest IF, which indicates greater recognition of relevant articles in these journals and the relatively high influence of these journals in the field. Both the Q1 and the Q2 journals and related publications had a relatively high number of citations, and the overall number of publications in the Q1 and Q2 journals was also high, indicating that JCR is also widely considered in publication and citation of research in this field, as in other fields such as immunology and psychiatry (44, 45). Notably, *Physiological Reviews* had a total of 901 citations and an average of 450.5 citations per document, both of which were at the top of the list of journals analysed, and this journal published two documents (40, 46), both reviews, which were the top two of the 10 most-cited articles and would explain the high number of citations (47). Moreover, this is the ideal journal in which to publish a review of this type, and the number of citations of these two documents in this journal will continue to increase as more research is conducted in this area.

As mentioned above, the two papers published in *Physiological Reviews*, “Calcitonin gene-related peptide: physiology and pathophysiology” (40) and “Neuronal control of skin function: the skin as a neuroimmunoendocrine organ” (46), had the greatest number of total citations, with “Calcitonin gene-related peptide: physiology and pathophysiology” (40) also having the highest average annual citations and the co-author Bunnett, Nigel W. of “Neuronal control of skin function: the skin as a neuroimmunoendocrine organ” (46) being the author with the highest total number of citations and average number of citations per document. In addition, “Skin acute wound healing: a comprehensive review” (48), published in 2019, already has 44.8 average annual citations only 4 years after publication, ranking third, indicating the high impact of all three papers. The earliest document was “Wound healing in denervated skin of rat” (33), published by Elfving G. in 1959 in *Annales Medicinae Experimentalis et Biologiae Fenniae*, although it was cited only four times, all before 1970. This was followed in 1964 by Elfving G.'s publication “Histochemical observations on wound healing in denervated + healthy rat skin” (34) in *Acta Pathologica et Microbiologica Scandinavica*, which was cited 13 times, all before 1985, and was the journal's second-earliest article and only article in this field. Neither this author nor these two articles are highly cited, probably because of how they were recorded, the author's country and institution, their accessibility to scholars, and their date of publication, but they are still cornerstones of the field.

An author's influence is judged not only by their total number of articles but also by a combination of their total number of citations, average number of citations per document, and collaboration with other expert authors (49). Gibran, Nicole S. cooperated with others the most and published the largest number of articles in the current field, mostly related to neuropeptides, microvascular endothelial cells, and diabetic wound healing (50–60), but did not rank in the top three for the total number of citations or the average number of citations per document. Bunnett, Nigel W., from *Emory University*, in contrast, ranked first in terms of total citations and average number of citations per document, but his total number of publications is only six, all focused on the function of neutral endopeptidase and substance P on skin wound healing (56, 60–62) and the neuroimmunoendocrine interaction of skin (8, 46), of which the study subjects showed a good concentration and two documents were co-authored by Gibran, Nicole S. (56, 60). Therefore, the influence of both authors in the field is high. Notably, the only Chinese author on the top 20 list, Yibing Wang from *Shandong University*, also published six articles in this field, but his total number of citations and average number of citations per document were the lowest, probably because research on the effect of CD271 on epidermal stem cells on wound healing is relatively niche (63–68), and considerable research results have been published in recent years. Furthermore, there are relatively few collaborations between China and *Shandong University* with other countries and institutions in this field.

The United States has the highest number of total publications, total citations, and collaborations with other countries. The *University of Washington*, with the highest number of publications is located in the United States, and the average publication year for the United States is 2010.18, while the average publication year for the *University of Washington* is 2005.13, indicating that American scholars and institutions are well established and firmly dominant in this field. China and India, the two developing countries on the top 10 list in terms of the number of publications, and *Shandong University*, the only developing country institution in the top 10 in terms of the number of publications, are at the bottom of the list of the total number of citations. The average date of publication of relevant documents of the two countries are after 2015, which indicates that China and India are at a relatively early stage in this field and does not yet have a strong influence. However, with the increase in Chinese and Indian research investment, increasing talent in this field, and strengthening of relevant international collaboration and cooperation, China, India, and their institutions and scholars are expected to become stars in the future.

The top 10 most frequently occurring author keywords cover the most basic, classic, and important research topics in this field. “Wound healing” and “skin” are common general keywords, while “neuropeptide,” “nerve growth factor,” and “substance P” are the main effectors of neurological factors. “Inflammation,” “keratinocytes,” “fibroblasts,” and “angiogenesis” are important participants in wound healing, and “diabetes mellitus” is one of the most frequent diseases with neuropathy-related wounds, which somewhat explains the accuracy of our search and the results of our analysis. The overlay and author keyword citation burst analyses provide a temporal and burst analysis of the hotspots in the relevant research area, respectively. Our results suggest that “stem cells,” including “mesenchymal stem cells” (41), and “immune cells,” including “macrophage cells” (69) and “mast cells” (70), are receiving increasing attention, with several studies published in the most recent years. In addition, “diabetic foot ulcers” are receiving increasing attention, possibly in relation to the increasing incidence of this kind of wound due to the ageing of the population and changes in lifestyle (71). Zhu et al. reported that substance P, combined with epidermal stem cells, promoted diabetic wound healing in 2016 (72). It is predicted that in the future, the modulation of stem cells by neurological factors and interventions for diabetic foot ulcers through neuroimmune modulation will be hot topics.

The present study has several limitations. First, we analysed only reviews and articles published in English in the WoSCC database. Documents in other languages, such as Chinese, were not included, which may lead to bias in the analysis results. Although the WoSCC database itself is large and comprehensive, using a single database may lead to the omission of data published in other sources, such as Medline, Scope, and Google Scholar. Future research could be extended to diverse databases. Second, we optimised our search methods as much as possible, but it is still possible that some literature was not included in the analysis. Third, because of the large amount of literature published worldwide on a daily basis, the conclusions obtained from our analysis using the search results in this paper are dynamic and temporal but can be followed up using the methods described in this paper to track the latest trends and research hotspots in the field.

## Conclusion

We used bibliometric methods to analyse reviews and articles related to the effect of skin innervation on wound healing published from 1959 to 2022 and included in the WoSCC database. During these 64 years, there has been a gradual increase in documents in this field, with the United States long taking the lead and interacting the most with other countries, while Chinese scholars have gradually begun to enter the field as rising stars. In the future, research on “stem cells,” “mesenchymal stem cells,” “immune cells,” and “diabetic foot ulcers” will be popular. The results of our bibliometric analysis in this field will help senior researchers identify new research directions and hotspots and will also help researchers who are new to the field to identify the trends and clarify the direction of development to prepare their knowledge base for future research.

## Data Availability

The original contributions presented in the study are included in the article/**Supplementary Material**, further inquiries can be directed to the corresponding author.
