# Supplementary material for: Research status and hot topics of the effects of skin innervation on wound healing from 1959 to 2022: A bibliometric analysis
Source: Front Surg. 2022 Oct 11;9:966375. doi: 10.3389/fsurg.2022.966375 (PMC9592856; doi:10.3389/fsurg.2022.966375)
Supplement: Supplementary file 1 [file DataSheet1.pdf]

Table S1. Annual publication count and annual total citations and annual average citations per document of the effect of skin innervation on wound healing

Year	Counts	Total cites	Cites per docs
1959	1	4	4.00
1964	1	13	13.00
1968	1	0	0.00
1984	1	72	72.00
1986	1	37	37.00
1991	2	255	127.50
1992	2	96	48.00
1994	2	123	61.50
1995	4	258	64.50
1996	5	403	80.60
1997	5	361	72.20
1998	10	659	65.90
1999	9	538	59.78
2000	6	182	30.33
2001	12	1035	86.25
2002	9	483	53.67
2003	12	952	79.33
2004	14	941	67.21
2005	9	398	44.22
2006	11	953	86.64
2007	14	652	46.57
2008	13	512	39.38
2009	8	351	43.88
2010	11	314	28.55
2011	12	586	48.83
2012	18	516	28.67
2013	22	646	29.36
2014	22	1437	65.32
2015	15	1048	69.87
2016	14	358	25.57
2017	17	305	17.94
2018	18	279	15.50
2019	9	416	46.22
2020	18	200	11.11
2021	25	61	2.44
2022	15	9	0.60

Table S2. Countries/Regions in each cluster based on co-authorship country network analysis

Cluster	Country/Region	Counts	Total link strength	Cites	Cites per docs	Average publication year
Cluster 1	France	14	5	807	57.64	2011.23
	Brazil	13	3	258	19.85	2013.00
	Canada	11	3	337	30.64	2013.64
	India	11	3	231	21.00	2015.00
Cluster 2	UK	42	18	2737	65.17	2007.00
	Germany	35	25	2409	68.83	2009.20
	Australia	11	2	185	16.82	2006.91
Cluster 3	USA	135	44	6608	48.95	2010.18
	China	34	6	274	8.06	2017.55
	South Korea	9	2	261	29.00	2012.89
Cluster 4	Italy	22	13	943	42.86	2010.91
	Sweden	9	5	556	61.78	2000.56
Cluster 5	Japan	38	13	1167	30.71	2012.00

Table S3. Institutions in each cluster based on co-authorship institution network analysis

Cluster	Institution	Counts	Total link strength	Cites	Cites per docs	Average publication year
Cluster 1	univ washington	16	11	1095	68.44	2005.13
	northwestern univ	6	4	251	41.83	2012.67
	univ calif san francisco	6	10	983	163.83	2001.00
	emory univ	5	7	536	107.20	1999.60
Cluster 2	harvard univ	8	2	489	61.13	2011.75
	univ coimbra	8	1	428	53.50	2013.63
	mit	7	1	353	50.43	2011.71
Cluster 3	univ manchester	13	5	286	22.00	2013.77
	univ munster	8	6	1131	141.38	2005.13
	univ miami	7	3	441	63.00	2017.86
Cluster 4	stanford univ	7	1	251	35.86	2012.71
	osaka univ	5	1	222	44.40	2009.80
Cluster 5	ucl	7	1	266	38.00	2002.57
	univ toronto	5	1	188	37.60	2012.20
Cluster 6	ohio state univ	5	0	226	45.20	2010.20
Cluster 7	shandong univ	7	0	29	4.14	2017.14

Cluster 8	univ calif irvine	6	0	554	92.33	2007.83
Cluster 9	univ kansas	6	0	286	47.67	2004.17
Cluster 10	univ limoges	5	0	116	23.20	2017.60
Cluster 11	univ melbourne	6	0	107	17.83	2004.50
Cluster 12	univ texas	5	0	277	55.40	2003.80

Table S4. Top 5 authors that published documents on the effects of skin innervation on wound healing of 3 stages (1991-2006, 2007-2014, 2015-2022)

Stage	Author	Counts	Total cites	Countries/Regions
Stage 1991-2006	Ansel, John C.	8	969	USA
	Olerud, John E.	7	743	USA
	Bunnett, Nigel W.	6	1009	USA
	Gibran, Nicole S.	6	401	USA
	Khalil, Zeinab G.	5	167	Australia
	Terenghi, Giorgio	5	163	UK
Stage 2007-2014	Carvalho, Eugenia	6	303	Portugal
	Satoh, Akira	6	336	Japan
	Gardiner, David M.	5	326	USA
	Gibran, Nicole S.	5	123	USA
	Moura, Liane I. F.	5	246	Portugal
Stage 2015-2022	Desmouliere, Alexis	5	116	France
	Calza, Laura	4	22	Italy
	Giardino, Luciana	4	22	Italy
	Girard, Dorothee	4	113	France
	Laverdet, Betty	4	113	France
	Pannella, Micaela	4	22	Italy
	Wang, Yibing	4	21	China

Table S5. Authors in each cluster based on co-authorship author network analysis

Cluster	Author	Counts	Total link strength	Cites	Cites per docs	Average publication year
Cluster 1	gibran, nicole s.	11	41	524	47.64	2005.09
	ansel, jc	8	27	969	121.13	2000.63
	olerud, je	7	31	743	106.14	2000.71

	bunnett, nigel w.	6	24	1009	168.17	2001.50
	muangman, pornprom	5	22	231	46.20	2006.00
	muffley, lara a.	5	18	255	51.00	2007.20
	isik, f. frank	4	18	233	58.25	2005.50
	usui, ml	4	18	312	78.00	2000.25
	luger, ta	3	3	395	131.67	2000.00
	scott, jeffrey r.	3	12	106	35.33	2008.00
	spenny, ml	3	16	174	58.00	2002.67
	sullivan, stephen r.	3	18	128	42.67	2004.33
	tamura, richard n.	3	12	81	27.00	2006.67
Cluster 2	desmouliere, alexis	6	12	159	26.50	2015.50
	paus, ralf	6	3	198	33.00	2014.17
	misery, laurent	5	12	205	41.00	2013.20
	berthod, francois	4	4	125	31.25	2016.00
	cheret, jeremy	4	6	99	24.75	2016.75
	girard, dorothee	4	12	113	28.25	2016.75
	laverdet, betty	4	12	113	28.25	2016.75
	lebonvallet, nicolas	3	8	111	37.00	2015.00
	matsuda, h.	3	1	297	99.00	2003.00
	peters, e. m. j.	3	2	161	53.67	2006.00
Cluster 3	wang, yibing	6	22	24	4.00	2016.50
	cao, yongqian	4	18	21	5.25	2016.75
	feng, yongqiang	3	9	13	4.33	2013.00
	li, xiaohong	3	15	21	7.00	2018.67
	ma, jiaxu	3	13	11	3.67	2020.00
	yin, siyuan	3	13	11	3.67	2020.00
	zhang, min	3	15	21	7.00	2018.67
	zhang, rui	3	11	6	2.00	2013.67
Cluster 4	luigi aloe	5	3	391	78.20	2006.40
	calza, laura	4	21	22	5.50	2020.75
	giardino, luciana	4	21	22	5.50	2020.75
	pannella, micaela	4	21	22	5.50	2020.75
	alastra, giuseppe	3	18	4	1.33	2021.00
	baldassarro, vito antonio	3	18	4	1.33	2021.00
	giuliani, alessandro	3	18	4	1.33	2021.00
	lorenzini, luca	3	18	4	1.33	2021.00
Cluster 5	carvalho, eugenia	8	15	428	53.50	2013.63
	moura, liane i. f.	5	11	246	49.20	2013.20

	veves, aristidis	5	9	621	124.20	2012.40
	cruz, maria teresa	4	8	129	32.25	2012.00
	leal, ermelindo c.	4	10	302	75.50	2014.00
	logerfo, frank w.	4	9	377	94.25	2012.00
	andersen, nicholas d.	3	6	266	88.67	2011.00
Cluster 6	akamatsu, hirohiko	3	12	45	15.00	2015.33
	hasegawa, seiji	3	12	45	15.00	2015.33
	matsunaga, kayoko	3	12	45	15.00	2015.33
	nakata, satoru	3	12	45	15.00	2015.33
	yagami, akiko	3	12	45	15.00	2015.33
Cluster 7	hasan, wohaib	3	6	142	47.33	2005.67
	liu, mx	3	4	162	54.00	2000.33
	mccarson, kenneth e.	3	5	124	41.33	2008.00
	rook, jerri m.	3	5	124	41.33	2008.00
	smith, pg	3	4	162	54.00	2000.33
Cluster 8	terenghi, giorgio	8	13	198	24.75	2004.38
	ferguson, mark william james	5	9	105	21.00	2010.40
	henderson, james	4	9	75	18.75	2010.00
	mcgrouther, d. a.	3	5	113	37.67	2002.67
	polak, jm	3	2	104	34.67	1996.33
Cluster 9	satoh, akira	7	10	346	49.43	2011.57
	gardiner, david m.	6	9	554	92.33	2007.83
	bryant, susan v.	5	8	520	104.00	2007.40
	makanae, aki	3	3	115	38.33	2015.33
Cluster 10	ferreira, lydia masako	4	9	56	14.00	2013.25
	hochman, bernardo	4	9	56	14.00	2013.25
	furtado, fabianne	3	8	51	17.00	2013.00
	liebano, richard eloin	3	6	48	16.00	2014.00
Cluster 11	gopal, anu	4	11	123	30.75	2015.00
	kant, vinay	4	11	123	30.75	2015.00
	kumar, dinesh	4	11	123	30.75	2015.00
	kumar, dhirendra	3	9	94	31.33	2014.00
Cluster 12	bigliardi, pl	3	6	142	47.33	2003.67
	bigliardi-qi, m	3	6	142	47.33	2003.67
	rufli, t	3	6	142	47.33	2003.67
Cluster 13	yannas, ioannis v.	4	6	198	49.50	2013.00
	so, peter t. c.	3	6	140	46.67	2015.67
	tzeranis, dimitrios s.	3	6	140	46.67	2015.67

Cluster 14	english, kb	3	3	61	20.33	1998.00
	tuckett, rp	3	3	61	20.33	1998.00
Cluster 15	fitzgerald, maria	5	3	285	57.00	2000.60
	reynolds, ml	3	3	231	77.00	1995.33
Cluster 16	monte-alto-costa, andrea	4	4	95	23.75	2011.50
	romana-souza, bruna	4	4	95	23.75	2011.50
Cluster 17	brain, s. d.	3	0	709	236.33	2004.00
Cluster 18	khalil, z.	6	0	170	28.33	2000.17
Cluster 19	ogawa, rei	3	0	82	27.33	2010.00
Cluster 20	paller, amy s.	3	0	28	9.33	2018.33
Cluster 21	werner, sabine	3	0	321	107.00	2005.00

Table S6. Author keywords in each cluster based on keyword network analysis

Cluster	Author keywords	Occurrences	Total link strength	Average publication year
Cluster 1	wound healing	107	221	2012.21
	diabetic foot ulcer	8	13	2016.38
	collagen	6	21	2012.33
	myofibroblast	6	15	2010.20
	pressure ulcer	6	12	2012.50
	nerve regeneration	5	20	2010.40
	sensory nerve	5	12	2001.80
	vascular endothelial growth factor	5	9	2015.60
	electrical stimulation	4	5	2010.50
	neurotensin	4	10	2013.25
	wound contraction	4	4	2011.00
	cutaneous innervation	3	8	1994.50
	mesenchymal stem cells	3	5	2016.33
Cluster 2	skin	31	89	2010.19
	inflammation	16	46	2009.00
	wound	11	34	2004.73
	pain	9	26	2012.11
	sensory neuron	9	32	2009.89
	cytokine	7	18	2013.57
	atopic dermatitis	3	12	2020.33

	fibrosis	3	10	2011.00
	itch	3	14	2020.67
	leukocyte	3	10	2009.33
	psoriasis	3	10	2015.67
	serotonin	3	4	2017.67
Cluster 3	fibroblast	18	61	2009.53
	regeneration	14	43	2010.21
	nerve	9	34	2009.33
	axolotl	6	20	2009.67
	dedifferentiation	6	27	2010.17
	blastema	5	11	2012.20
	epidermis	5	12	2007.80
	tgf beta	5	9	2009.20
	activin	4	13	2008.00
	bone morphogenetic protein	4	11	2011.75
	limb regeneration	4	8	2012.25
	limb	3	14	2008.33
Cluster 4	cd271 (p75ntr)	11	30	2013.00
	epidermal stem cell	5	18	2017.60
	stem cell	5	6	2016.00
	burn	4	11	2017.00
	migration	4	9	2001.25
	proliferation	4	8	2007.75
	skin wound healing	4	10	2018.75
	tyrosine kinase a	4	14	2008.00
	cell proliferation	3	13	2010.33
	hair follicle	3	12	2006.67
	peripheral nerve	3	3	2014.00
Cluster 5	diabetes mellitus	21	51	2012.67
	macrophage	10	26	2012.70
	denervation	9	25	2011.22
	transcutaneous electric nerve stimulation	7	11	2009.83
	rat	6	14	2006.17
	chronic wound	5	10	2017.67
	microcirculation	4	9	1999.75
	neuropathy	4	8	2005.00
	beta-adrenoceptor	3	8	2013.33
	nitric oxide	3	4	2008.33

Cluster 6	substance p	25	66	2008.75
	calcitonin gene related peptide	12	33	2006.08
	capsaicin	7	12	2002.14
	innervation	7	14	2013.00
	scar	5	18	2013.80
	reinnervation	4	12	2008.25
	cutaneous	3	8	2005.67
	injury	3	6	2004.00
	pgp 9.5	3	7	2002.00
Cluster 7	neuropeptide	34	90	2009.23
	nerve growth factor	26	62	2006.81
	angiogenesis	14	29	2013.79
	neurotrophin	5	18	2006.20
	endothelial cell	4	14	2005.50
	wound repair	4	12	2009.75
	neurite outgrowth	3	3	2008.33
	neuroinflammation	3	11	2004.33
Cluster 8	neurogenic inflammation	7	22	2005.71
	mast cell	4	10	2014.00
	mouse	3	13	2011.00
	nerve fiber	3	10	2012.00
	neuromediators	3	5	2017.33
	stress	3	6	2009.67
	sympathetic nervous system	3	6	2004.00
Cluster 9	keratinocyte	19	52	2007.33
	keloid	3	15	2012.67
	tissue engineering	3	7	2010.33
	trigeminal trophic syndrome	3	3	2008.67
Cluster 10	cutaneous wound healing	3	4	2015.00
	knockout mice	3	9	2009.33
